# RNA-Redesign: a web server for fixed-backbone 3D design of RNA

**DOI:** 10.1093/nar/gkv465

**Published:** 2015-05-11

**Authors:** Joseph D. Yesselman, Rhiju Das

**Affiliations:** 1 Biochemistry Department, Stanford University, Stanford, CA 94305, USA; 2 Physics Department, Stanford University, Stanford, CA 94305, USA

## Abstract

RNA is rising in importance as a design medium for interrogating fundamental biology and for developing therapeutic and bioengineering applications. While there are several online servers for design of RNA secondary structure, there are no tools available for the rational design of 3D RNA structure. Here we present RNA-Redesign (http://rnaredesign.stanford.edu), an online 3D design tool for RNA. This resource utilizes fixed-backbone design to optimize the sequence identity and nucleobase conformations of an RNA to match a desired backbone, analogous to fundamental tools that underlie rational protein engineering. The resulting sequences suggest thermostabilizing mutations that can be experimentally verified. Further, sequence preferences that differ between natural and computationally designed sequences can suggest whether natural sequences possess functional constraints besides folding stability, such as cofactor binding or conformational switching. Finally, for biochemical studies, the designed sequences can suggest experimental tests of 3D models, including concomitant mutation of base triples. In addition to the designs generated, detailed graphical analysis is presented through an integrated and user-friendly environment.

## INTRODUCTION

Engineered RNA and RNA/protein complexes are offering new routes for deciphering genetic regulation and for the eventual rational design of diagnostics and therapeutics (1–5). These designed or repurposed molecules harness RNA's ability to adopt complex 3D shapes, to perform catalysis (6), to guide pairing to DNA or RNA (7), and to change shapes in response to cellular and viral molecules (8–10). Unfortunately, development of RNA's potential as a design medium is hindered by a lack of available computational tools, necessitating time-consuming selection methods, experimental expertise and/or trial-and-error refinement. While there are numerous online servers for design of RNA secondary structure (11,12), packages aiming for the rational design of 3D RNA structure currently require expert intuition, installation of complex software or both (13,14). Here we present a tool for rational design of new RNA molecules that is available online through a fast web server.

The RNA-Redesign web server (http://rnaredesign.stanford.edu) utilizes the rna_design application in the Rosetta 3 framework to optimize the primary sequence of an RNA molecule to match a given phosphate-sugar backbone, analogous to the fixed-backbone design tools that underlie most approaches to rational protein engineering (15). In addition to designing new RNA folds, this approach can suggest functional constraints, stabilize specific 3D RNA motifs and suggest mutations to validate predicted folds. In addition to outputting 3D models of designed sequences with the lowest Rosetta effective energy, RNA-Redesign also outputs a graphical weblogo outlining the nucleotide preferences at each sequence. Also, RNA-Redesign reports a graph of the native sequence recovery, reflecting how often the input nucleotide reappears at each sequence position, which can facilitate discrimination of positions that are likely to be sequence-constrained for structural or non-structural reasons.

## METHOD OVERVIEW

The main goal of RNA-Redesign is to find sequence variants compatible with a backbone structure. It has been tested for three tasks: (i) stabilizing an existing structure relative to natural counterparts, (ii) highlighting differences in natural and computationally preferred sequences that might suggest functional constraints and (iii) designing mutations that allow tests of a predicted fold via compensatory rescue. The application returns sequence designs within minutes for RNA structures with lengths under 20 residues. Long input structures (up to 100 nt) are also accepted but require longer computational times; for these cases, the users may wish to provide an e-mail for notification of job completion. Examples of each application are presented below.

RNA-Redesign takes an RNA 3D structure in the form of a PDB-formatted file. The file must contain backbone and sugar heavy atoms and may contain nucleobase atoms (which will be replaced during design). RNA-Redesign uses the Rosetta packer algorithm, well-developed for protein design (16) to sample the base identity and side-chain torsions. For RNA, these routines optimize new base conformations that best fit into the phosphate-sugar backbone. Ideal glycosidic torsion angles (χ) for each sugar pucker are generated by averaging over all RNA residues with that sugar pucker in the ribosome crystal structure (PDB ID: 1JJ2) (17). During the side-chain packing, χ values of −1, −$\frac{1}{2}$, 0, $\frac{1}{2}$ and +1 standard deviations away from ideal are sampled at each nucleotide, analogous to sampling of side-chain torsions for protein side chains. For purine nucleotides (guanosine and adenosine), *syn* rotamers for χ appear in natural RNA molecules and are therefore also sampled. The placement of the 2′-OH hydrogen is also simultaneously optimized with the base rotamers; the torsion angle defined by the C3′-C2′-O2′-HO2′ atoms are sampled at six torsion angles (−140°, −80°, −20°, +40°, +100° and +160°). The optimization of the nucleotide side-chain conformation and identity is carried out simultaneously at all residues by simulated anneling; the packing energy computations are accelerated by precomputation of all rotamer–rotamer pairwise energies. To sample a more detailed landscape of the sequence preference at each nucleotide position, the algorithm performs several independent separate nucleobase packing runs (10 by default, but up to 100 can be scheduled by the user).

## WEB SERVER

**Figure 1.**
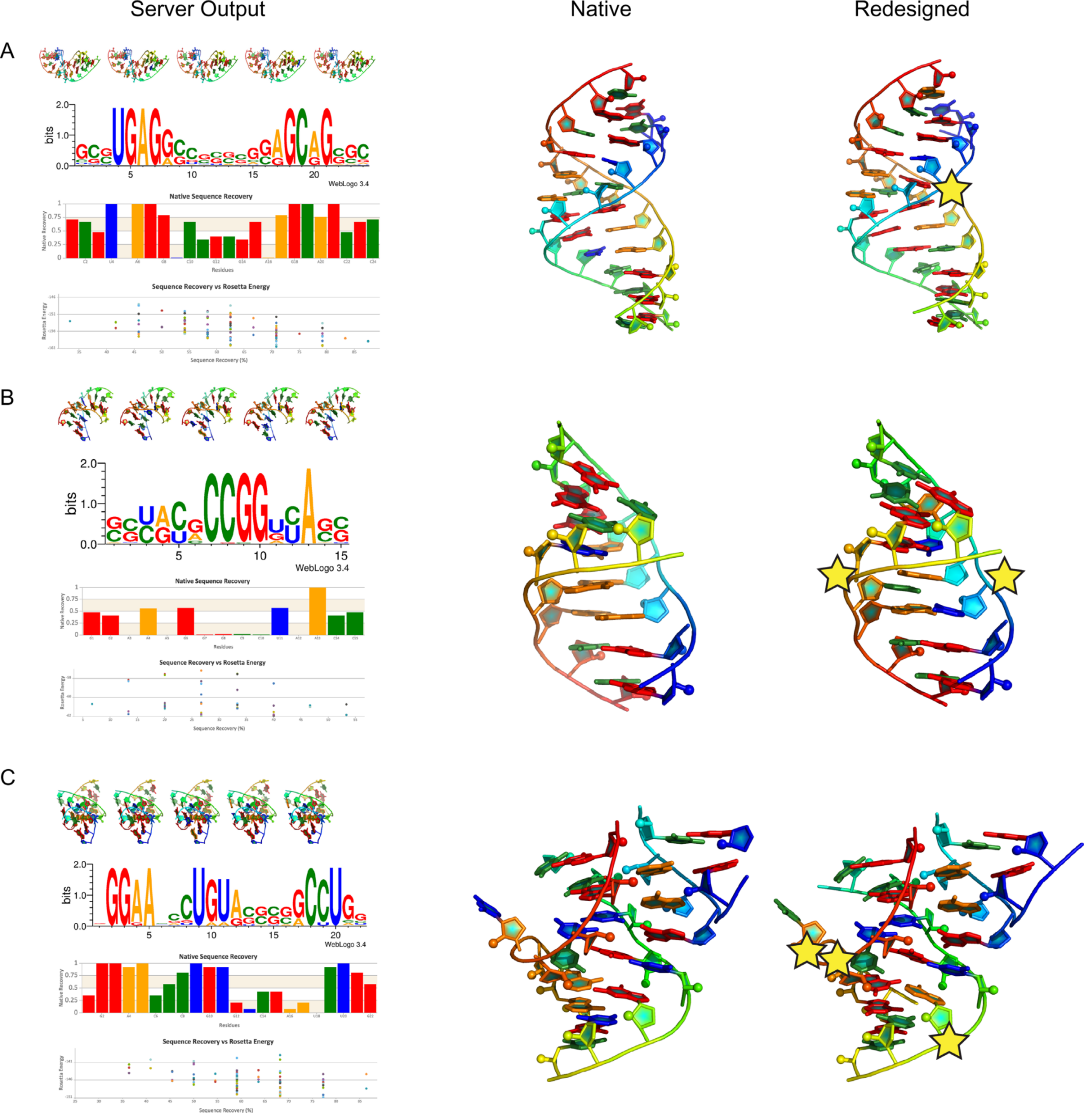
Stars indicate key mutations suggested by RNA-Redesign. (**A**) SRP domain IV RNA (PDB ID: 1LNT) contains highly conserved AC base pairs that RNA-Redesign mutates to stabilize the RNA. (**B**) J4/5 domain of the *Tetrahymena* ribozyme (PDB ID: 1GID), contains two AA sheared base pairs that are mutated to an AU and an AC base pair. (**C**) A proposed 3D model for the GGAA tetraloop/tetraloop-receptor tertiary contact. RNA-Redesign finds three residues that are predicted to be mutatable without loss of function.

The Redesign server uses the CherryPy (http://www.cherrypy.org/) framework based on Python (https://www.python.org/) for basic web services and data management. For client-side programming, we coded the web pages in the HTML5 (http://www.w3.org/TR/html5/) standard, with jQuery (http://jquery.com) for interactive user-interface components and Bootstrap (http://getbootstrap.com/) for styling. On the server side, each job is generated with an ID that is assigned to a static link to the wait/results page in the form http://redesign.stanford.edu/result/JOB_ID. The data stay on the server for one month. The results are parsed by Python and rendered onto the web page, as shown in Figure 1. RNA-Redesign supports most of the widely used web browsers including Google Chrome, Mozilla Firefox, Apple Safari and Microsoft Internet Explorer.

The results page (Figure 1) has four sections. At the top is a 3D representation of the top five sequences in the desired fold. Each image is generated by performing a ray trace in PyMOL (http://www.pymol.org/) using an in-house PyMOL coloring and graphics scheme (freely available at https://github.com/DasLab/pymol_daslab). Next, using the WebLogo3 python package (https://pypi.python.org/pypi/weblogo), a weblogo is generated to display the sequence propensity at each sequence location. Coloring is static with guanosine, cytidine, adenosine, uridine as red, green, gold and blue respectively. Finally, the native sequence recovery is plotted using CanvasJS (http://canvasjs.com) an interactive plotting interface in JavaScript. This is the likelihood of recovering the native sequence at each position. This plot can be informative because a nucleotide that is rarely recovered through RNA-Redesign but is naturally conserved may not be critical for structural stability and is therefore a candidate for other functional roles such as protein binding or catalysis. At the bottom, there is an interactive scatter plot, comparing Rosetta energy to sequence recovery. Users interested in examining specific designs can use this plot as a guide to see which designs are most favorable and which give the highest sequence recovery.

To demonstrate the versatility of the RNA-Redesign server, we have selected three design problems. First, we examined the signal recognition particle (SRP) domain IV RNA (PDB ID: 1LNT), which has a conserved set of functional A–C base pairs (18). Utilizing RNA-Redesign, we determined that one of A–C base pairs could be mutated to an A–G base pair, which yields a lower Rosetta energy (Figure 1A). A similar mutation was observed in prior studies and was demonstrated to yield a stabilized mutant (15). Next, we applied RNA-Redesign to the J4/5 motif from the P4-P6 domain of group I *Tetrahymena* ribozyme (PDB ID: 1GID), which forms contacts in the ribozyme's core (19). J4/5 is comprised of a conserved G–U base pair above an unpaired A and two sheared A–A base pairs. Mutational profiling and structural studies of the full ribozyme have revealed that both the G–U and A–A base pairs, while not forming any tertiary contacts in the isolated P4-P6 domain, form contacts between the P4-P6 domain and the full ribozyme's catalyic core (20). RNA-Redesign mutates the A–A base pairs into an A–U Watson–Crick base pair and an A–C Hoogsteen base pair (Figure 1B), additionally suggesting that the A–A base pairs are not necessary for stabilizing the motif but are instead conserved due to structural roles or catalytic roles in the context of the full ribozyme. Lastly, we submitted a possible model for a GGAA tetraloop/tetraloop-receptor developed by Jaeger *et al.* (21) (Figure 1C). This model was generated by a new *de novo* 3D structure prediction software currently in development by our lab (22). RNA-Redesign suggests several mutations that do not appear to affect the overall fold—binding affinity measurements for variants with single and multiple mutations in combination could validate or disprove this proposed fold. For example, a G–A (Figure 1C, residues marked with a star) pair is often redesigned to an A–G pair; this switch is known experimentally to have a negligible effect on the stability of the motif (21). Other mutations, such as a C-G/C base triple occasionally mutated by RNA-redesign to a U-A/A triple, are predicted to be tolerated if made concomitantly; their test would provide evidence for this de novo modeled structure.

## SUMMARY

We developed RNA-Redesign (freely available at http://rnaredesign.stanford.edu), an online server for RNA 3D design. RNA-Redesign uses the Rosetta 3 framework to design and to optimize the primary sequence of RNA with a given 3D backbone structure. These results can identify functional constraints and can suggest mutations that may stabilize or test the structures of a specific 3D RNA motif. It is our hope that RNA-Redesign can contribute to the growing development of RNA-based technology for biochemical and bioengineering applications.
